# The chromosomal genome sequence of the maze coral, *Meandrina meandrites* (Linnaeus, 1758) (Scleractinia: Meandrinidae) and its associated microbial metagenome sequences

**DOI:** 10.12688/wellcomeopenres.27184.1

**Published:** 2026-07-28

**Authors:** Julia Marie Stewart, Mónica Medina, Andrew Bruckner, Lisa May, Zachary J. Moffitt, Jose Victor Lopez, Cheryl M. Woodley, Sebastian Metz, Michael Sweet, Nina Pruzinsky, Graeme Oatley, Elizabeth Sinclair, Eerik Aunin, Noah Gettle, Camilla Santos, Michael Paulini, Haoyu Niu, Victoria McKenna, Rebecca O’Brien, Wellcome Sanger Institute Tree of Life Management, Samples and Laboratory team, Wellcome Sanger Institute Scientific Operations: Sequencing Operations, Wellcome Sanger Institute Tree of Life Core Informatics team, EBI Aquatic Symbiosis Genomics Data Portal Team, Aquatic Symbiosis Genomics Project Leadership

**Affiliations:** 1 Marine Science Department, College of Natural and Health Sciences, University of Hawai‘i at Hilo, Hilo, HI, USA; 2 Department of Ecology and Evolutionary Biology, University of California Los Angeles, Los Angeles, California, USA; 3 Florida Keys National Marine Monument, Key Largo, FL, USA; 4 NOAA National Center for Coastal Ocean Science, Charleston, SC, USA; 5 National Coral Reef Institute, Department of Biological Sciences, Nova Southeastern University, Dania Beach, FL, USA; 6 Aquatic Research Facility, Nature-based Solutions Research Cen, University of Derby, Derby, England, UK; 7 University Corporation for Atmospheric Research (UCAR), Boulder, CO, USA; 8 NOAA Ocean Exploration Affiliate, Silver Spring, MD, USA; 9 Tree of Life Programme, Wellcome Sanger Institute, Hinxton, England, UK

**Keywords:** Meandrina meandrites, maze coral, genome sequence, chromosomal, Scleractinia, microbial metagenome assembly

## Abstract

We present a genome assembly from a specimen of *Meandrina meandrites* (maze coral; Cnidaria; Anthozoa; Scleractinia; Meandrinidae). The genome sequence has a total length of 551.16 megabases. Most of the assembly (99.25%) is scaffolded into 14 chromosomal pseudomolecules. The mitochondrial genome has also been assembled, with a length of 17.2 kilobases. Gene annotation of this assembly by Ensembl identified 30 464 protein-coding genes. We recovered two bins from the metagenome data.

## Species taxonomy

Eukaryota; Opisthokonta; Metazoa; Eumetazoa; Cnidaria; Anthozoa; Hexacorallia; Scleractinia; Vacatina; Meandrinidae; *Meandrina*; *Meandrina meandrites* (Linnaeus, 1758) (NCBI:txid51056).

## Background

*Meandrina meandrites* (Linnaeus, 1758), commonly known as the Maze coral or Brain coral, is a species of zooxanthellate stony coral belonging to the family Meandrinidae (Cnidaria: Anthozoa: Hexacorallia). *M. meandrites* is typically found in the western Atlantic, from Bermuda to Florida, throughout the Caribbean, and as far south as Brazil. It prefers shallow waters averaging depths of 1–12 m. At the higher and lower latitudes of its biogeographic range, such as locations like Bermuda and Curaçao, these corals usually inhabit depths to 30 m ( Bak & Luckhurst, 1980). The morphology of this species is often encrusting, with hemispherical colonies found in low-light areas ( Sandeman, 2008). Thick theca and large calyces form cliff-and-valley patterns that provide protection for long, large tentacles ( Johnson & Sebens, 1993). While growing, they lay down calcium bands during the day, which can lead to an average growth of 11 μm per day ( Sandeman, 2008). The trabeculae, or vertical aragonite banding, of *M. meandrites* are readily visualised by microscope and can be used to track seasonal growth ( Sandeman, 2008).

*M. meandrites* exhibits both sexual hermaphroditic reproduction through broadcast spawning and asexual reproduction ( Baird *et al.*, 2009; Pinzón & Weil, 2011). Studies of pre-spawning gametogenesis and post-spawning tissue histology have identified species-specific reproductive patterns ( Pinzón & Weil, 2011). Until recently, sexual recruits were observed only in the field, though artificial spawning events have been successfully conducted in aquaria mimicking the lunar and environmental cycles of the Florida Reef Tract ( O’Neil *et al.*, 2021).

*M. meandrites* can be host to several different types of organisms, from protists to small crabs. This species engages in a mutualistic relationship with symbiotic dinoflagellates (zooxanthellae) in the family Symbiodiniaceae ( LaJeunesse *et al.*, 2018), which provide the coral with essential nutrients through photosynthesis. *Breviolum* spp. are the most common symbionts reported from Barbados, Belize, and Curaçao ( Finney *et al.*, 2010; Frade *et al.*, 2016), whereas in Colombia, different colonies may host species of *Breviolum*, *Cladocopium*, or both ( Grajales & Sánchez, 2016). In a study from Curaçao, the symbiont density was reported to be 7.9 ± 4.4 × 10^6 cells cm ^−2^, which is greater than *Porites astreoides* and *Siderastraea siderea*, two other dominant corals in the Caribbean ( Frade *et al.*, 2016). The authors also observed that the *M. meandrites* tissue thickness appeared greater than the other two major reef-builders, which may explain why the Symbiodiniaceae cells are so abundant ( Frade *et al.*, 2016). In the mesenterial filaments of *M. meandrites*, histological and molecular studies have identified *Gemmocystis cylindrus* Upton and Peters, 1986 as a common apicomplexan ( Kwong *et al.*, 2021; Upton & Peters, 1986). While many known organisms found in *M. meandrites* tissue or mucus are non-parasitic, this coral can be infected with the gall crab, *Troglocarcinus corallicola* Verrill, 1908, as documented in Curaçao and Brazil ( van der Meij, 2014).

Like most corals, *M. meandrites* is susceptible to coral bleaching. However, *M. meandrites* is able to regenerate its tissue faster than other common reef-building corals ( Meesters & Bak, 1993). Some studies have also shown that *M. meandrites* have been able to survive, recover and proliferate after thermal stress events. In 2005, more than 80 individual coral colonies had bleached, with a very low mortality rate ( Steiner & Kerr, 2008). A year later, more colonies were found than the previous year, increasing coral cover from 18% to 22%, with only about a quarter reported as bleached ( Steiner & Kerr, 2008). Its documented ability to recover from thermal stress makes *M. meandrites* a compelling coral to study at the molecular level.

Stony Coral Tissue Loss Disease (SCTLD) ( NOAA Florida Keys National Marine Sanctuary, 2018) is a contagious infectious disease representing a significant threat to coral reefs in the Caribbean. *M. meandrites* is highly susceptible to SCTLD, which was first identified in 2014 off the south-east coast of Florida and has since spread throughout the region, affecting numerous coral species ( Precht *et al.*, 2016). The disease causes significant tissue loss in affected colonies, leading to mortality and significantly reduced coral cover on reefs. After the emergence and rapid spread of SCTLD, the IUCN Red List of Threatened Species listed *M. meandrites* as Critically Endangered in 2021 ( Crabbe *et al.*, 2022).

Although the mitochondrial genome sequence of *M. meandrites* was recently published ( Baeza & Rosales, 2025), omic studies of *M. meandrites* are limited. The *M. meandrites* microbiome has been characterised along the Florida Reef Tract using 16S rDNA gene amplicons or metagenomes of apparently healthy and SCTLD-infected corals ( Heinz *et al.*, 2024; Rosales *et al.*, 2022, 2023). With increasing frequency of metagenomic studies, and given the current Red List status of *M. meandrites*, high-quality whole genome data are timely to help investigate the effects of SCTLD on this vulnerable species. The specimen used for this purpose was collected near Key West in the Florida Keys National Marine Sanctuary. With its expansive range across the Caribbean, the ability to grow to depths of 30 m, the ability to regenerate tissue, and its potential thermal tolerance, *M. meandrites* is a compelling species to study at the genomic level.

## Methods

### Sample acquisition

**Figure 1.**
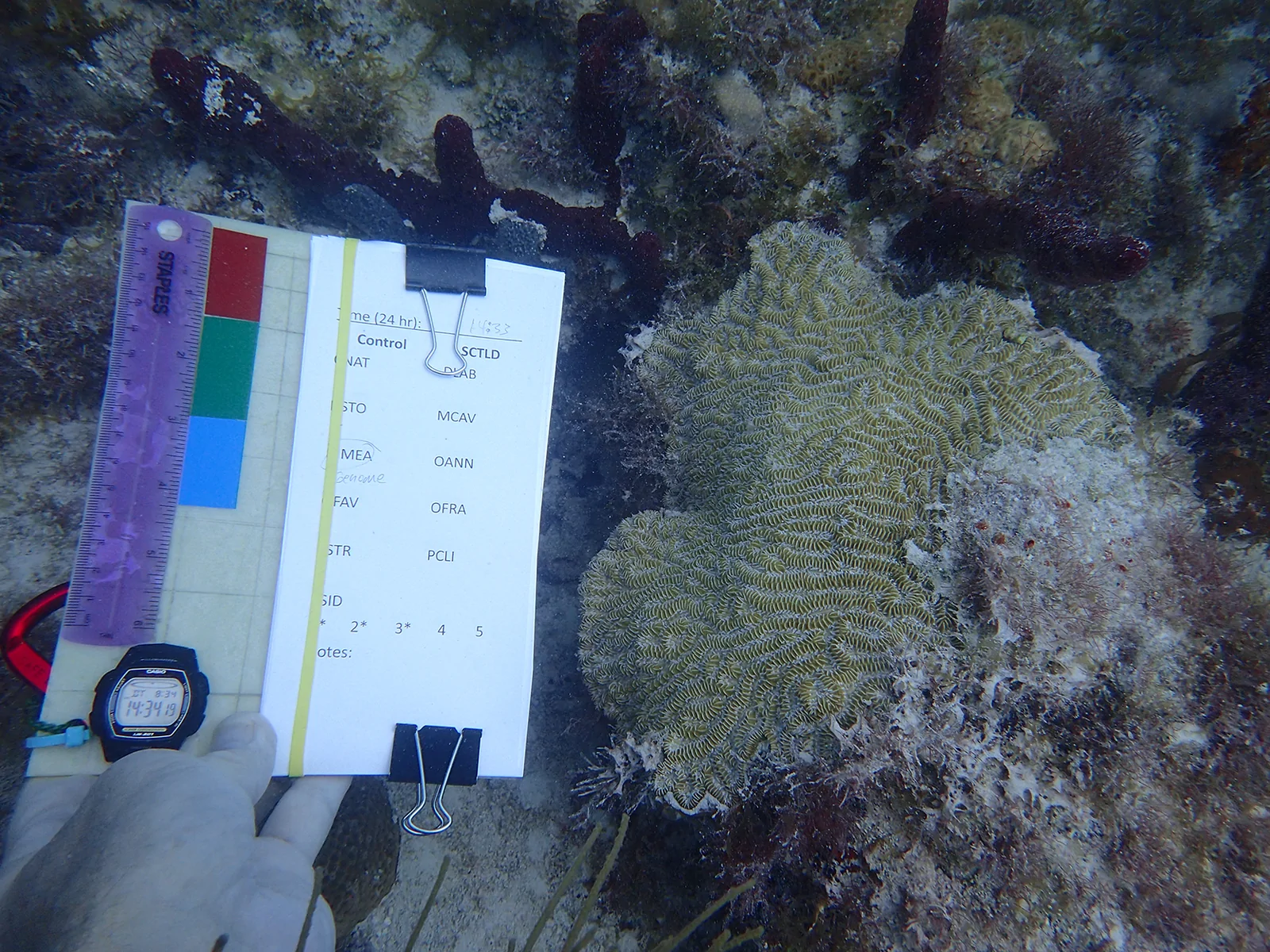
*Meandrina meandrites* colony from which a fragment was collected on 17 September 2019 from The key west national marine monument by andrew bruckner.

A *M. meandrites* adult coral colony fragment (specimen ID NSU0022001, ToLID jaMeaMean2), was collected on 2019-09-17 in the Florida Keys National Marine Sanctuary off Key West, Florida, USA (latitude 24.46, longitude −81.98) where no apparent SCTLD-affected corals were found. A visual health assessment was conducted and the colony was photographed ( Figure 1). The jaMeaMean2 specimen was also used for RNA sequencing.

Part of the colony was removed from the reef by hammer and chisel under the permit number FNMKS-2019-069 awarded to Cheryl Woodley. The live colony was transported in clean seawater with an air bubbler to NOAA Hollings Marine Lab where it was maintained until shipped to the Wellcome Sanger Institute (WSI) for genome sequencing.

### Nucleic acid extraction

Protocols for high molecular weight (HMW) DNA extraction developed at the WSI Tree of Life Core Laboratory are available on protocols.io ( Howard *et al.*, 2025). The jaMeaMean2 sample was weighed and triaged to determine the appropriate extraction protocol. The sample was homogenised using the cryogenic bead beating protocol. HMW DNA was extracted using the Manual MagAttract protocol.

Two different DNA preparations were used for sequencing: one for low-input and one for ultra-low input sequencing. For the first, DNA was sheared into an average fragment size of 12–20 kb following the Megaruptor®3 for LI PacBio protocol. For the second, we used centrifuge-mediated fragmentation to produce DNA fragments in the 8–10 kb range, following the Covaris g-TUBE protocol for ultra-low input (ULI). Sheared DNA was purified by manual SPRI (solid-phase reversible immobilisation). The concentration of the sheared and purified DNA was assessed using a Nanodrop spectrophotometer and Qubit Fluorometer using the Qubit dsDNA High Sensitivity Assay kit. Fragment size distribution was evaluated by running the sample on the FemtoPulse system.

RNA was extracted from modular colony tissue of the jaMeaMean2 specimen in the Tree of Life Laboratory at the WSI using the RNA Extraction: Automated MagMax™ *mir*Vana protocol. The RNA concentration was assessed using a Nanodrop spectrophotometer and a Qubit Fluorometer using the Qubit RNA Broad-Range Assay kit. Analysis of the integrity of the RNA was done using the Agilent RNA 6000 Pico Kit and Eukaryotic Total RNA assay.

### PacBio HiFi library preparation and sequencing

Library preparation and sequencing were performed at the WSI Scientific Operations core. For low-input PacBio sequencing, libraries were prepared using the SMRTbell Prep Kit 3.0 (Pacific Biosciences, California, USA), following the manufacturer’s instructions. The kit includes reagents for end repair/A-tailing, adapter ligation, post-ligation SMRTbell bead clean-up, and nuclease treatment. Size selection and clean-up were performed using diluted AMPure PB beads (Pacific Biosciences). DNA concentration was quantified using a Qubit Fluorometer v4.0 (ThermoFisher Scientific) and the Qubit 1X dsDNA HS assay kit. Final library fragment size was assessed with the Agilent Femto Pulse Automated Pulsed Field CE Instrument (Agilent Technologies) using the gDNA 55 kb BAC analysis kit.

Prior to library preparatio for ULI sequencing, the DNA was fragmented to ~10 kb. Ultra-low-input (ULI) libraries were prepared using the PacBio SMRTbell® Express Template Prep Kit 2.0 and gDNA Sample Amplification Kit. Samples were normalised to 20 ng DNA. Single-strand overhang removal, DNA damage repair, and end-repair/A-tailing were performed according to the manufacturer’s instructions, followed by adapter ligation. A 0.85× pre-PCR clean-up was carried out with Promega ProNex beads.

The DNA was evenly divided into two aliquots for dual PCR (reactions A and B), both following the manufacturer’s protocol. A 0.85× post-PCR clean-up was performed with ProNex beads. DNA concentration was measured using a Qubit Fluorometer v4.0 (Thermo Fisher Scientific) with the Qubit HS Assay Kit, and fragment size was assessed on an Agilent Femto Pulse Automated Pulsed Field CE Instrument (Agilent Technologies) using the gDNA 55 kb BAC analysis kit. PCR reactions A and B were then pooled, ensuring a total mass of ≥500 ng in 47.4 μl.

The pooled sample underwent another round of DNA damage repair, end-repair/A-tailing, and hairpin adapter ligation. A 1× clean-up was performed with ProNex beads, followed by DNA quantification using the Qubit and fragment size analysis using the Agilent Femto Pulse. Size selection was performed on the Sage Sciences PippinHT system, with target fragment size determined by Femto Pulse analysis (typically 4–9 kb). Size-selected libraries were cleaned with 1.0× ProNex beads and normalised to 2 nM before sequencing.

The sample was sequenced using the Sequel IIe system (Pacific Biosciences, California, USA). The concentration of the library loaded onto the Sequel IIe was in the range 40–135 pM. SMRT Link software, a PacBio web-based workflow manager, was used to set up and monitor the run, and to perform primary and secondary analysis of the data upon completion.

### Hi-C

***Sample preparation and crosslinking***

The Hi-C sample was prepared from 20–50 mg of frozen modular colony tissue of the jaMeaMean2 sample using the Arima-HiC v2 kit (Arima Genomics). Following the manufacturer’s instructions, tissue was fixed and DNA crosslinked using TC buffer to a final formaldehyde concentration of 2%. The tissue was homogenised using the Diagnocine Power Masher-II. Crosslinked DNA was digested with a restriction enzyme master mix, biotinylated, and ligated. Clean-up was performed with SPRISelect beads before library preparation. DNA concentration was measured with the Qubit Fluorometer (Thermo Fisher Scientific) and Qubit HS Assay Kit. The biotinylation percentage was estimated using the Arima-HiC v2 QC beads.

***Hi-C library preparation and sequencing***

Biotinylated DNA constructs were fragmented using a Covaris E220 sonicator and size selected to 400–600 bp using SPRISelect beads. DNA was enriched with Arima-HiC v2 kit Enrichment beads. End repair, A-tailing, and adapter ligation were carried out with the NEBNext Ultra II DNA Library Prep Kit (New England Biolabs), following a modified protocol where library preparation occurs while DNA remains bound to the Enrichment beads. Library amplification was performed using KAPA HiFi HotStart mix and a custom Unique Dual Index (UDI) barcode set (Integrated DNA Technologies). Depending on sample concentration and biotinylation percentage determined at the crosslinking stage, libraries were amplified with 10–16 PCR cycles. Post-PCR clean-up was performed with SPRISelect beads. Libraries were quantified using the AccuClear Ultra High Sensitivity dsDNA Standards Assay Kit (Biotium) and a FLUOstar Omega plate reader (BMG Labtech).

Prior to sequencing, libraries were normalised to 10 ng/μL. Normalised libraries were quantified again to create equimolar and/or weighted 2.8 nM pools. Pool concentrations were checked using the Agilent 4200 TapeStation (Agilent) with High Sensitivity D500 reagents before sequencing. Sequencing was performed using paired-end 150 bp reads on the Illumina NovaSeq 6000.

### RNA library preparation and sequencing

Libraries were prepared using the NEBNext® Ultra II Directional RNA Library Prep Kit for Illumina (New England Biolabs), following the manufacturer’s instructions. Poly(A) mRNA was isolated from 100 ng of total RNA using oligo (dT) beads, converted to cDNA, and uniquely indexed; 14 PCR cycles were performed. Libraries were prepared with an intended fragment size of 100–300 bp. Libraries were quantified, normalised, and pooled equimolarly to a final concentration of 2.8 nM before dilution to 150 pM for loading. Sequencing was carried out on the Illumina NovaSeq 6000, generating paired-end reads.

### Genome assembly

Prior to assembly of the PacBio HiFi reads, a database of *k*-mer counts ( *k* = 31) was generated from the filtered reads using FastK. GenomeScope2 ( Ranallo-Benavidez *et al.*, 2020) was used to analyse the *k*-mer frequency distributions, providing estimates of genome size, heterozygosity, and repeat content.

The HiFi reads were assembled using Hifiasm ( Cheng *et al.*, 2021) with the --primary option. Haplotypic duplications were identified and removed using purge_dups ( Guan *et al.*, 2020). The Hi-C reads ( Rao *et al.*, 2014) were mapped to the primary contigs using bwa-mem2 ( Vasimuddin *et al.*, 2019), and the contigs were scaffolded in YaHS ( Zhou *et al.*, 2023) with the --break option for handling potential misassemblies. The scaffolded assemblies were evaluated using Gfastats ( Formenti *et al.*, 2022), BUSCO ( Manni *et al.*, 2021) and MERQURY.FK ( Rhie *et al.*, 2020).

The mitochondrial genome was assembled using MitoHiFi ( Uliano-Silva *et al.*, 2023). The MitoHiFi reference was the mitochondrial genome of *Diploastrea heliopora* (NC_060802.1).

### Assembly curation

The assembly was decontaminated using the Assembly Screen for Cobionts and Contaminants ( ASCC) pipeline. TreeVal was used to generate the flat files and maps for use in curation. Manual curation was conducted primarily in PretextView and HiGlass ( Kerpedjiev *et al.*, 2018). Scaffolds were visually inspected and corrected as described by Howe *et al*. (2021). Manual corrections included 20 breaks, 20 joins, and removal of 12 haplotypic duplications. This reduced the scaffold count by 50.4%, increased the scaffold N50 by 1.4%, and reduced the total assembly length by 2.8%. The curation process is described at sanger-tol/curation-resources . PretextSnapshot was used to generate a Hi-C contact map of the final assembly.

### Assembly quality assessment

The Merqury.FK tool ( Rhie *et al.*, 2020) was run in a Singularity container ( Kurtzer *et al.*, 2017) to evaluate *k*-mer completeness and assembly quality for the primary and alternate haplotypes using the *k*-mer database ( *k* = 31) computed prior to genome assembly. The analysis outputs included assembly QV scores and completeness statistics.

The genome was analysed using the BlobToolKit pipeline, a Nextflow implementation of the earlier Snakemake version ( Challis *et al.*, 2020). PacBio reads were aligned to the assembly using minimap2 ( Li, 2018) and processed with SAMtools ( Danecek *et al.*, 2021) to generate coverage tracks. The pipeline runs BUSCO ( Manni *et al.*, 2021) using lineages identified from the NCBI Taxonomy ( Schoch *et al.*, 2020). For the three basal BUSCO lineages, eukaryota, bacteria and archaea, BUSCO genes are aligned to the UniProt Reference Proteomes database ( Bateman *et al.*, 2023) using DIAMOND BLASTp ( Buchfink *et al.*, 2021). The genome assembly is divided into chunks based on the density of BUSCO genes from the closest taxonomic lineage, and each chunk is aligned to the UniProt Reference Proteomes database using DIAMOND BLASTx. Sequences without DIAMOND BLASTx hits are extracted using seqtk and aligned to the NT database using BLASTn ( Altschul *et al.*, 1990). The outputs are consolidated into a BlobDir for visualisation in BlobToolKit. The BlobToolKit pipeline was developed using nf-core tooling ( Ewels *et al.*, 2020) and MultiQC ( Ewels *et al.*, 2016), with containerisation through Docker ( Merkel, 2014) and Singularity ( Kurtzer *et al.*, 2017).

## Metagenome assembly

The metagenome assembly was generated using MetaMDBG ( Benoit *et al.*, 2024). The resulting bin sets of each binning algorithm were optimised and refined using MAGScoT ( Rühlemann *et al.*, 2022). PROKKA ( Seemann, 2014) was used to identify tRNAs and rRNAs in each bin, CheckM ( Parks *et al.*, 2015) (checkM_DB release 2015-01-16) was used to assess bin completeness/contamination, and GTDB-Tk ( Chaumeil *et al.*, 2022) (GTDB release 214) was used to taxonomically classify bins. Taxonomic replicate bins were identified using dRep ( Olm *et al.*, 2017) with default settings (95% ANI threshold). All bins were assessed for quality and categorised as metagenome-assembled genomes (MAGs) if they met the following criteria: contamination ≤5%, presence of 5S, 16S, and 23S rRNA genes, at least 18 unique tRNAs, and either ≥90% completeness or ≥ 50% completeness with fully circularised chromosomes ( Bowers *et al.*, 2017). Bins that did not meet these thresholds, or were identified as taxonomic replicates of MAGs, were retained as ‘binned metagenomes’ provided they had ≥50% completeness and ≤ 10% contamination.

## Genome sequence report

### Sequence data

**Figure 2.**
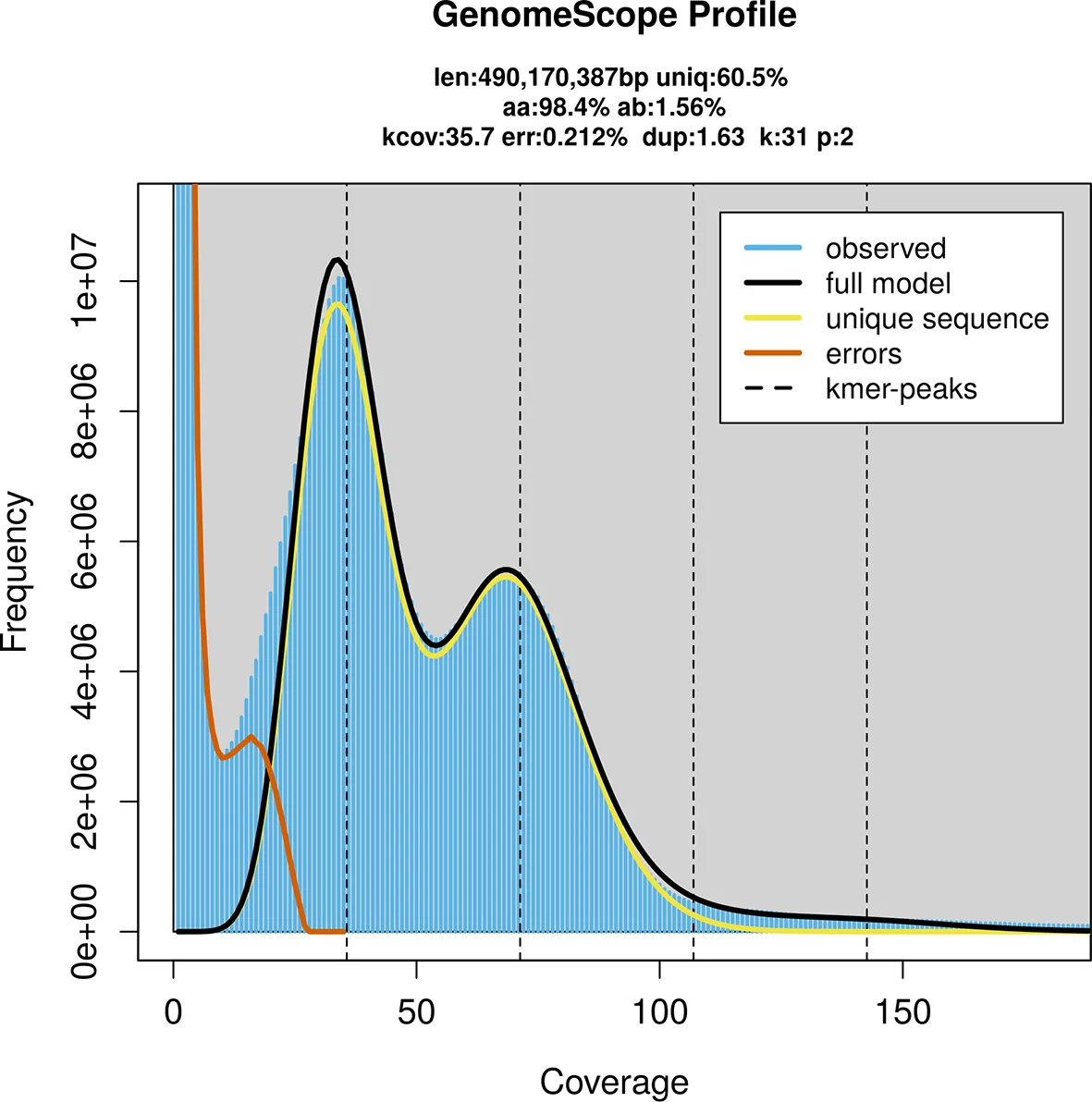
Frequency distribution of *k*-mers generated using GenomeScope2. The plot shows observed and modelled *k*-mer spectra, providing estimates of genome size, heterozygosity, and repeat content based on unassembled sequencing reads.

PacBio sequencing of the *Meandrina meandrites* specimen generated 38.92 Gb (gigabases) from 3.80 million reads, which were used to assemble the genome. GenomeScope2.0 analysis estimated the haploid genome size at 491.31 Mb, with a heterozygosity of 1.56% and repeat content of 39.53% ( Figure 2). These estimates guided expectations for the assembly. Based on the estimated genome size, the sequencing data provided approximately 74× coverage. Hi-C sequencing produced 13.72 Gb from 45.42 million reads, which were used to scaffold the assembly. RNA sequencing data were also generated and are available in public sequence repositories. Table 1 summarises the specimen and sequencing details.

**Table 1.** Specimen and sequencing data for *meandrina meandrites* (BioProject PRJEB69492).

Platform	PacBio HiFi	Hi-C	RNA-seq
**ToLID**	jaMeaMean2	jaMeaMean2	jaMeaMean2
**Specimen ID**	NSU0022001	NSU0022001	NSU0022001
**BioSample (source individual)**	SAMEA12928865	SAMEA12928865	SAMEA12928865
**BioSample (tissue)**	SAMEA12928866	SAMEA12928873	SAMEA12928867
**Tissue**	modular colony	modular colony	modular colony
**Instrument**	Sequel IIe	Illumina NovaSeq 6000	Illumina NovaSeq 6000
**Run accessions**	ERR12303928; ERR12303929	ERR12318574	ERR12321244
**Read count total**	3.80 million reads	45.42 million read pairs	34.78 million read pairs
**Base count total**	38.92 Gb	13.72 Gb	10.50 Gb

### Assembly statistics

The primary haplotype was assembled, and contigs corresponding to an alternate haplotype were also deposited in INSDC databases. The final assembly has a total length of 551.16 Mb in 56 scaffolds, with 138 gaps, and a scaffold N50 of 39.93 Mb ( Table 2).

**Table 2.** Genome assembly data for *meandrina meandrites.*

Genome assembly	Primary assembly
**Assembly name**	jaMeaMean2.1
**Assembly accession**	GCA_963693305.1
**Alternate haplotype accession**	GCA_963693325.1
**Assembly level**	chromosome
**Span (Mb)**	551.16
**Number of chromosomes**	14
**Number of contigs**	194
**Contig N50**	7.63 Mb
**Number of scaffolds**	56
**Scaffold N50**	39.93 Mb
**Organelles**	Mitochondrial genome: 17.20 kb

The scaffold count follows the NCBI assembly statistics and excludes organelle assemblies, which are reported separately where present.

**Figure 3.**
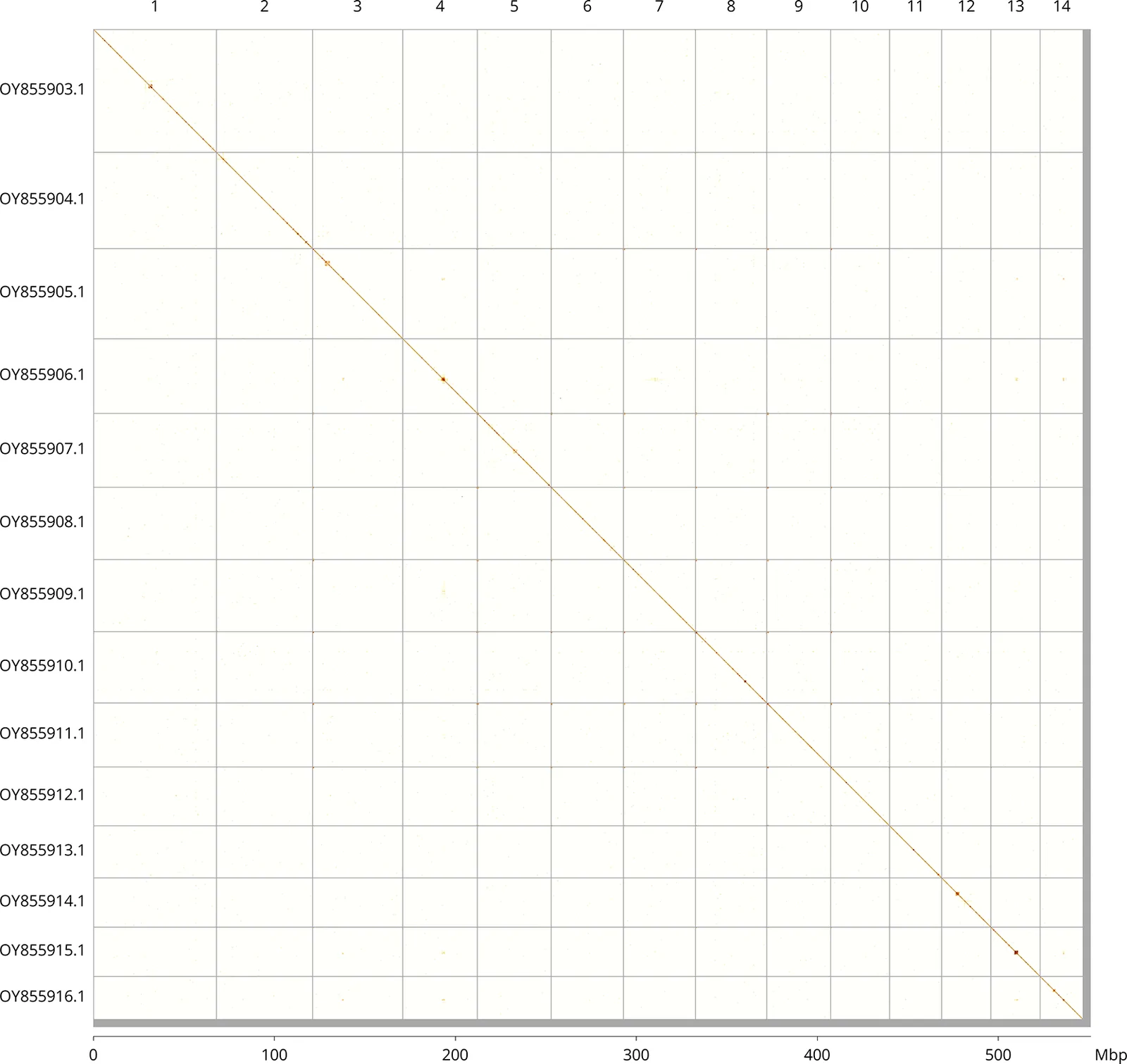
Hi-C contact map of the *Meandrina meandrites* genome assembly. Assembled chromosomes are shown in order of size and labelled along the axes, with a megabase scale shown below. The plot was generated using PretextSnapshot.

Most of the assembly sequence (99.25%) was assigned to 14 chromosomal-level scaffolds. These chromosome-level scaffolds, confirmed by Hi-C data, are named according to size ( Figure 3; Table 3).

**Table 3.** Chromosomal pseudomolecules in the primary genome assembly of *meandrina meandrites* jaMeaMean2.

INSDC accession	Molecule	Length (Mb)	GC%
OY855903.1	1	68.06	39
OY855904.1	2	53.09	39
OY855905.1	3	49.81	39
OY855906.1	4	41.34	39.50
OY855907.1	5	40.70	39.50
OY855908.1	6	39.93	39.50
OY855909.1	7	39.88	39.50
OY855910.1	8	39.38	39.50
OY855911.1	9	35.33	39.50
OY855912.1	10	32.53	39.50
OY855913.1	11	28.73	39.50
OY855914.1	12	27.25	39
OY855915.1	13	27.10	39
OY855916.1	14	23.88	39

The mitochondrial genome was also assembled (length 17.2 kb, OY855917.1). This sequence is included as a contig in the multifasta file of the genome submission and as a standalone record.

### Assembly quality metrics

**Figure 4.**
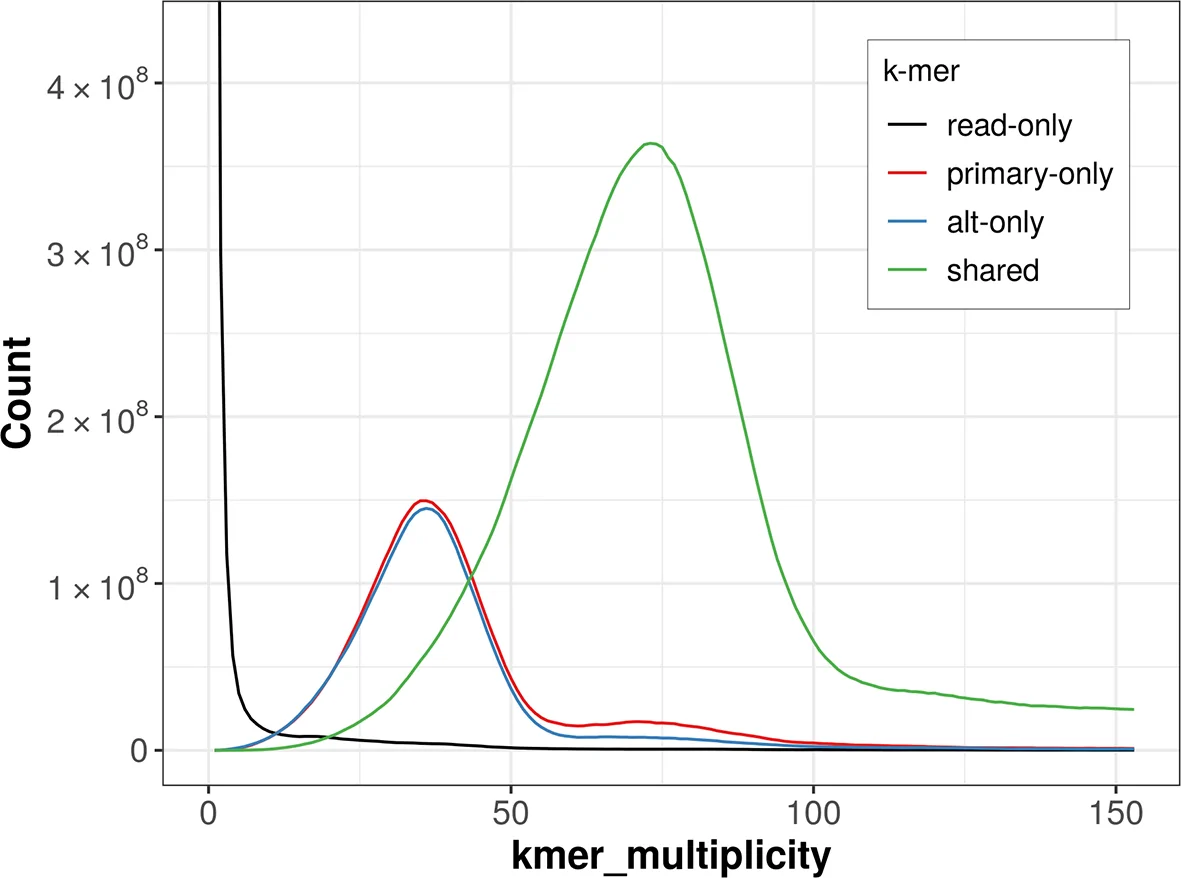
Evaluation of *k*-mer completeness using MerquryFK. This plot illustrates the recovery of *k*-mers from the original read data in the final assemblies. The horizontal axis represents *k*-mer multiplicity, and the vertical axis shows the number of *k*-mers. The black curve represents *k*-mers that appear in the reads but are not assembled. The green curve corresponds to *k*-mers shared by both haplotypes, and the red and blue curves show *k*-mers found only in one of the haplotypes.

The combined primary and alternate assemblies achieve an estimated QV of 61.4. The *k*-mer completeness is 75.79% for the primary assembly, 73.91% for the alternate haplotype, and 97.85% for the combined assemblies ( Figure 4).

**Figure 5.**
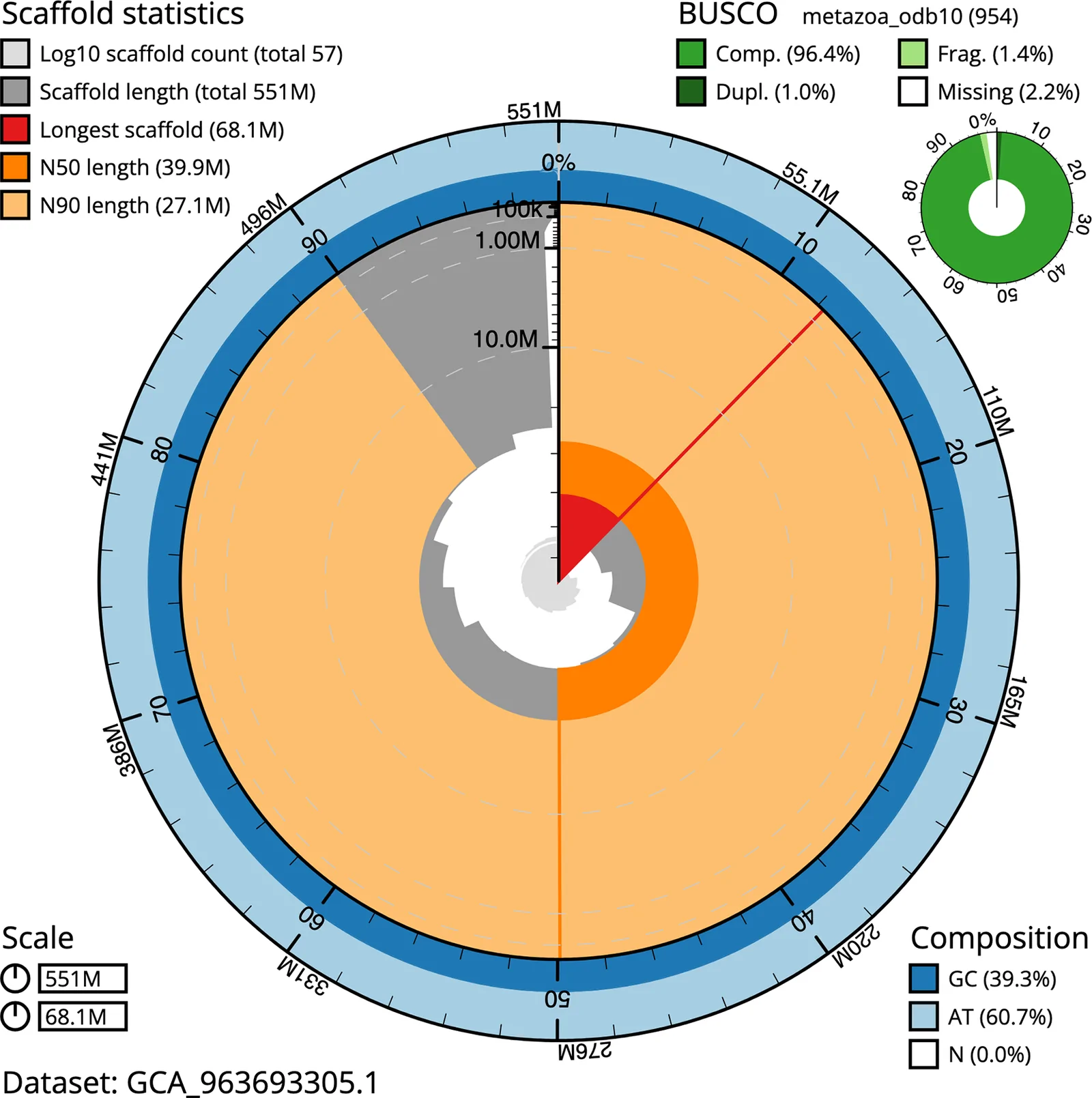
Assembly metrics for jaMeaMean2.1. The BlobToolKit snail plot provides an overview of assembly metrics and BUSCO gene completeness. The circumference represents the length of the whole genome sequence, and the main plot is divided into 1 000 bins around the circumference. The outermost blue tracks display the distribution of GC, AT, and N percentages across the bins. Scaffolds are arranged clockwise from longest to shortest and are depicted in dark grey. The longest scaffold is indicated by the red arc, and the deeper orange and pale orange arcs represent the N50 and N90 lengths. A light grey spiral at the centre shows the cumulative scaffold count on a logarithmic scale. A summary of complete, fragmented, duplicated, and missing BUSCO genes in the metazoa_odb10 set is presented at the top right. An interactive version of this figure can be accessed on the BlobToolKit viewer.

**Figure 6.**
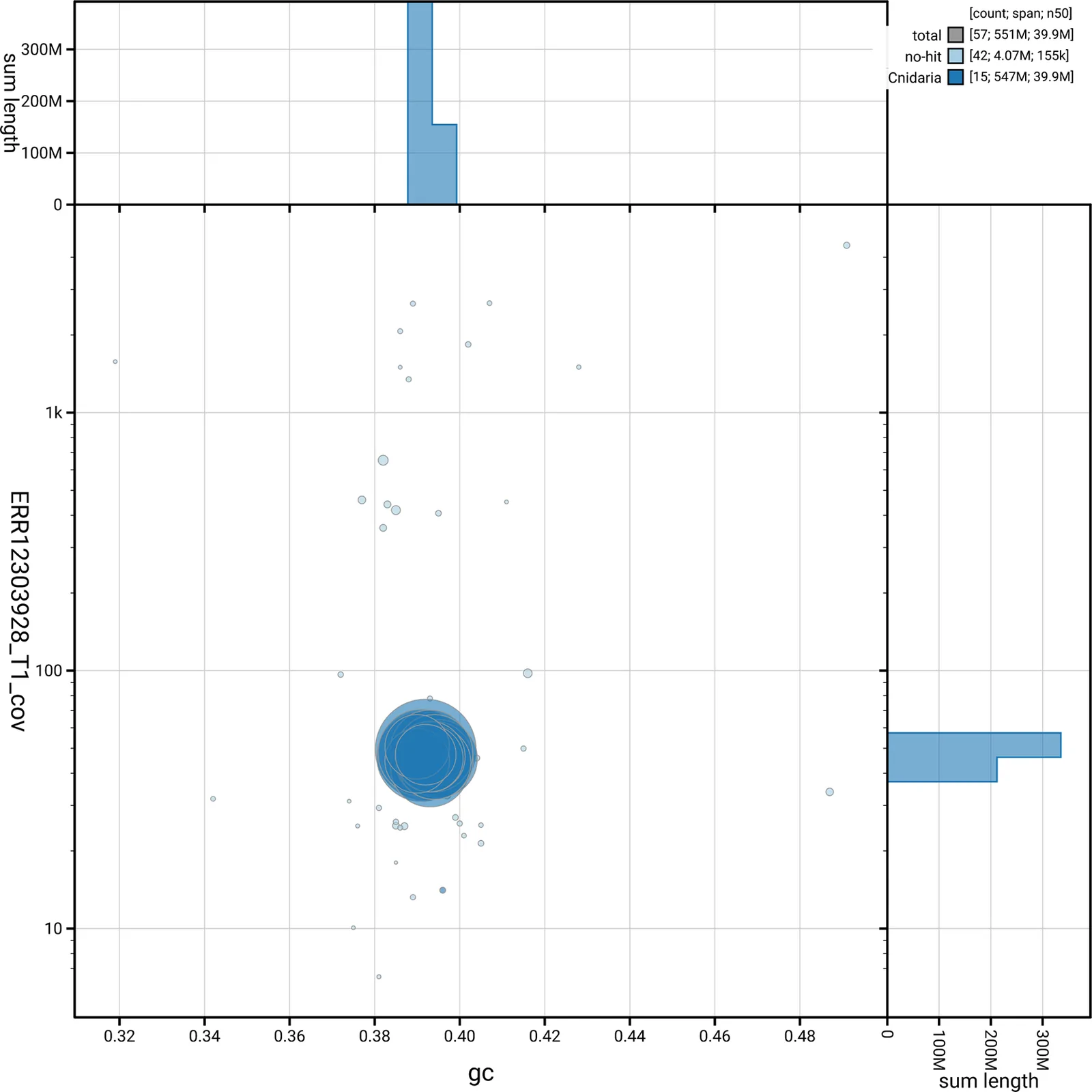
BlobToolKit blob plot for jaMeaMean2.1. The plot shows base coverage (vertical axis) and GC content (horizontal axis). The circles represent scaffolds, with the size proportional to scaffold length and the colour representing phylum membership. The histograms along the axes display the total length of sequences distributed across different levels of coverage and GC content. An interactive version of this figure is available on the BlobToolKit viewer.

BUSCO v.5.5.0 analysis using the metazoa_odb10 reference set ( *n* = 954) identified 96.4% of the expected gene set (single = 95.4%, duplicated = 1.0%). The snail plot in Figure 5 summarises the scaffold length distribution and other assembly statistics for the primary assembly. The blob plot in Figure 6 shows the distribution of scaffolds by GC proportion and coverage.

Table 4 lists the assembly metric benchmarks adapted from Rhie *et al.* (2021) and the Earth BioGenome Project Report on Assembly Standards January 2026. The EBP metric, calculated for primary assembly, is **6.C.Q61**, meeting the recommended 6.C.Q40 reference standard.

**Table 4.** Earth Biogenome Project summary metrics for the *Meandrina meandrites* assembly.

Measure	Value	Benchmark
EBP summary (primary)	6.C.Q61	6.C.Q40
Contig N50 length	7.63 Mb	≥ 1 Mb
Scaffold N50 length	39.93 Mb	= chromosome N50
Consensus quality (QV)	Primary: 61.0; alternate: 61.9; combined: 61.4	≥ 40
*k*-mer completeness	Primary: 75.79%; alternate: 73.91%; combined: 97.85%	≥ 95%
BUSCO	C:96.4% [S:95.4%, D:1.0%], F:1.4%, M:2.2%, n:954	S > 90%; D < 5%
Percentage of assembly assigned to chromosomes	99.25%	≥ 90%

*Notes:* The EBP summary uses log10(Contig N50); chromosome-level (C) or log10(Scaffold N50); Q (Merqury QV). BUSCO: C = complete; S = single-copy; D = duplicated; F = fragmented; M = missing; n = orthologues.

### Genome annotation report

The *Meandrina meandrites* genome assembly (GCA_963693305.1) was annotated by Ensembl at the European Bioinformatics Institute (EBI). This annotation includes 57 445 transcribed mRNAs from 30 464 protein-coding and 14 289 non-coding genes. The average transcript length is 7 092.63 bp, with an average of 1.28 coding transcripts per gene and 5.63 exons per transcript. Further details of this annotation are available from the Ensembl annotation page.

## Metagenome report

We recovered two bins from the metagenome assembly, which did not meet the criteria for MAGs. Table 5 summarises the taxa and quality of the metagenome bins.

**Table 5.** Quality metrics and taxonomic assignments of the binned metagenomes.

NCBI taxon	Taxid	GTDB taxonomy	Quality	Size (bp)	Contigs	Circular	Mean coverage	Completeness (%)	Contamination (%)
Alphaproteobacteria bacterium	1913988	Alphaproteobacteria	MEDIUM	2583228	1	Yes	17.62	98.92	0
Ichthyobacteriaceae bacterium	2774601	Bacteroidia	MEDIUM	1556950	62	No	2.59	67.52	0.12

**Table 6.** Software versions and sources used for *Meandrina meandrites.*

Software	Version	Source
BLAST	2.14.0	ftp://ftp.ncbi.nlm.nih.gov/blast/executables/blast+/
BlobToolKit	4.3.3	https://github.com/blobtoolkit/blobtoolkit
BUSCO	5.5.0; 5.4.3	https://gitlab.com/ezlab/busco
bwa-mem2	2.2.1	https://github.com/bwa-mem2/bwa-mem2
CheckM	2015-01-16	https://ecogenomics.github.io/CheckM/
DIAMOND	2.1.8	https://github.com/bbuchfink/diamond
dRep	3.4.0	https://github.com/MrOlm/drep
ete3	3.1.3	http://etetoolkit.org
fasta_windows	0.2.4	https://github.com/tolkit/fasta_windows
FastK	1.1	https://github.com/thegenemyers/FASTK
GenomeScope2.0	2.0.1	https://github.com/tbenavi1/genomescope2.0
Gfastats	1.3.6	https://github.com/vgl-hub/gfastats
GTDB-Tk	1.2.1	https://github.com/Ecogenomics/GTDBTk
Hifiasm	0.16.1	https://github.com/chhylp123/hifiasm
HiGlass	1.13.4	https://github.com/higlass/higlass
MAGScoT	1.0.0	https://github.com/ikmb/MAGScoT
MerquryFK	1.1.0-c1	https://github.com/thegenemyers/MERQURY.FK
MetaMDBG	Pre-release	https://github.com/GaetanBenoitDev/metaMDBG
Minimap2	2.24-r1122	https://github.com/lh3/minimap2
MitoHiFi	2	https://github.com/marcelauliano/MitoHiFi
MultiQC	1.14; 1.17 and 1.18	https://github.com/MultiQC/MultiQC
Nextflow	23.04.1	https://github.com/nextflow-io/nextflow
PretextSnapshot	0.0.5	https://github.com/sanger-tol/PretextSnapshot
PretextView	1.0.3	https://github.com/sanger-tol/PretextView
Prokka	1.14.5	https://github.com/tseemann/prokka
purge_dups	1.2.3	https://github.com/dfguan/purge_dups
samtools	1.16.1; 1.17	https://github.com/samtools/samtools
sanger-tol/ascc	0.1.0	https://github.com/sanger-tol/ascc
sanger-tol/blobtoolkit	0.3.0	https://github.com/sanger-tol/blobtoolkit
sanger-tol/curationpretext	1.4.2	https://github.com/sanger-tol/curationpretext
Seqtk	1.4-r122	https://github.com/lh3/seqtk
Singularity	3.9.0	https://github.com/sylabs/singularity
TreeVal	1.0.0	https://github.com/sanger-tol/treeval
YaHS	1.1a.2	https://github.com/c-zhou/yahs

## Author information

Contributors are listed at the following links:

• Members of the Wellcome Sanger Institute Tree of Life Management, Samples and Laboratory team
• Members of Wellcome Sanger Institute Scientific Operations – Sequencing Operations
• Members of the Wellcome Sanger Institute Tree of Life Core Informatics team
• Members of the EBI Aquatic Symbiosis Genomics Data Portal Team
• The Aquatic Symbiosis Genomics Project leadership

## Wellcome sanger institute – Legal and governance

The materials that have contributed to this genome note have been supplied by a Tree of Life collaborator. The Wellcome Sanger Institute employs a process whereby due diligence is carried out proportionate to the nature of the materials themselves, and the circumstances under which they have been/are to be collected and provided for use. The purpose of this is to address and mitigate any potential legal and/or ethical implications of receipt and use of the materials as part of the research project, and to ensure that in doing so we align with best practice wherever possible.

The overarching areas of consideration are:

• Ethical review of provenance and sourcing of the material
• Legality of collection, transfer and use (national and international)

Each transfer of samples is undertaken according to a Research Collaboration Agreement or Material Transfer Agreement entered into by the Tree of Life collaborator, Genome Research Limited (operating as the Wellcome Sanger Institute) and in some circumstances other Tree of Life collaborators.

## Data Availability

European Nucleotide Archive: Meandrina meandrites (maze coral). Accession number PRJEB69492; BioProject URL: https://identifiers.org/ena.embl/PRJEB69492. The genome sequence is released openly for reuse. The *Meandrina meandrites* genome sequencing initiative is part of the Aquatic Symbiosis Genomics Project (PRJEB43743) and the Sanger Institute Tree of Life Programme (PRJEB43745). All raw sequence data and the assembly have been deposited in INSDC databases. Raw data and assembly accession identifiers are reported in Tables 1 and 2. Production code used in genome assembly at the WSI Tree of Life is available at https://github.com/sanger-tol . Table 6 lists software versions used in this study.

## References

[ref1] Altschul SF Gish W Miller W : Basic Local Alignment Search Tool. *J. Mol. Biol.* 1990;215(3):403–410. 2231712 10.1016/S0022-2836(05)80360-2

[ref2] Baeza JA Rosales SM : The complete mitochondrial genome of the “maze” coral *Meandrina meandrites* (Scleractinia: Vacatina: Meandrinidae). *Mitochondrial DNA Part A.* 2025;35:154–161. 40481811 10.1080/24701394.2025.2504422

[ref3] Baird AH Guest JR Willis BL : Systematic and biogeographical patterns in the reproductive biology of scleractinian corals. *Annu. Rev. Ecol. Evol. Syst.* 2009;40:551–571. 10.1146/annurev.ecolsys.110308.120220

[ref4] Bak RPM Luckhurst BE : Constancy and change in coral reef habitats along depth gradients at Curaçao. *Oecologia.* 1980;47(2):145–155. 10.1007/BF00346812 28309463

[ref5] Bateman A Martin M-J Orchard S : UniProt: The Universal Protein Knowledgebase in 2023. *Nucleic Acids Res.* 2023;51(D1):D523–D531. 36408920 10.1093/nar/gkac1052 PMC9825514

[ref6] Benoit G Raguideau S James R : High-quality metagenome assembly from long accurate reads with metaMDBG. *Nat. Biotechnol.* 2024;42:1378–1383. 38168989 10.1038/s41587-023-01983-6 PMC11392814

[ref7] Bowers RM Kyrpides NC Stepanauskas R : Minimum information about a single amplified genome (MISAG) and a metagenome-assembled genome (MIMAG) of bacteria and archaea. *Nat. Biotechnol.* 2017;35(8):725–731. 28787424 10.1038/nbt.3893 PMC6436528

[ref8] Buchfink B Reuter K Drost H-G : Sensitive protein alignments at tree-of-life scale using DIAMOND. *Nat. Methods.* 2021;18(4):366–368. 33828273 10.1038/s41592-021-01101-x PMC8026399

[ref9] Challis R Richards E Rajan J : BlobToolKit – interactive quality assessment of genome assemblies. *G3: Genes, Genomes, Genetics.* 2020;10(4):1361–1374. 32071071 10.1534/g3.119.400908 PMC7144090

[ref10] Chaumeil P-A Mussig AJ Hugenholtz P : GTDB-Tk v2: Memory friendly classification with the genome taxonomy database. *Bioinformatics.* 2022;38(23):5315–5316. 36218463 10.1093/bioinformatics/btac672 PMC9710552

[ref11] Cheng H Concepcion GT Feng X : Haplotype-resolved *de novo* assembly using phased assembly graphs with Hifiasm. *Nat. Methods.* 2021;18(2):170–175. 33526886 10.1038/s41592-020-01056-5 PMC7961889

[ref12] Crabbe J Kennedy E Banaszak A : *Meandrina meandrites.* The IUCN Red List of Threatened Species. 2022;2022:e.T133224A165781180. Reference Source

[ref13] Danecek P Bonfield JK Liddle J : Twelve years of SAMtools and BCFtools. *GigaScience.* 2021;10(2):giab008. 33590861 10.1093/gigascience/giab008 PMC7931819

[ref14] Ewels P Magnusson M Lundin S : MultiQC: Summarize analysis results for multiple tools and samples in a single report. *Bioinformatics.* 2016;32(19):3047–3048. 27312411 10.1093/bioinformatics/btw354 PMC5039924

[ref15] Ewels PA Peltzer A Fillinger S : The nf-core framework for community-curated bioinformatics pipelines. *Nat. Biotechnol.* 2020;38(3):276–278. 32055031 10.1038/s41587-020-0439-x

[ref16] Finney JC Pettay DT Sampayo EM : The relative significance of host–habitat, depth, and geography on the ecology, endemism, and speciation of coral endosymbionts in the genus *Symbiodinium.* *Microb. Ecol.* 2010;60(1):250–263. 20502891 10.1007/s00248-010-9681-y

[ref17] Formenti G Abueg L Brajuka A : Gfastats: Conversion, evaluation and manipulation of genome sequences using assembly graphs. *Bioinformatics.* 2022;38(17):4214–4216. 35799367 10.1093/bioinformatics/btac460 PMC9438950

[ref18] Frade PR Schwaninger V Glasl B : Dimethylsulfoniopropionate in corals and its interrelations with bacterial assemblages in coral surface mucus. *Environ. Chem.* 2016;13:252–265. 10.1071/EN15023

[ref19] Grajales A Sánchez JA : Holobiont assemblages of dominant coral species ( *Symbiodinium* types and coral species) shape Caribbean reef community structure. *Revista de la Academia Colombiana de Ciencias Exactas, Físicas y Naturales.* 2016;40(155):300–311. 10.18257/raccefyn.294

[ref20] Guan D McCarthy SA Wood J : Identifying and removing haplotypic duplication in primary genome assemblies. *Bioinformatics.* 2020;36(9):2896–2898. 31971576 10.1093/bioinformatics/btaa025 PMC7203741

[ref21] Heinz JM Lu J Huebner LK : Novel metagenomics analysis of stony coral tissue loss disease. *G3: Genes, Genomes, Genetics.* 2024;14(8):jkae137. 38900914 10.1093/g3journal/jkae137 PMC11304949

[ref22] Howard C Denton A Jackson B : On the path to reference genomes for all biodiversity: Lessons learned and laboratory protocols created in the Sanger Tree of Life core laboratory over the first 2000 species. *bioRxiv.* 2025. 10.1101/2025.04.11.648334 PMC12548527 41129326

[ref23] Howe K Chow W Collins J : Significantly improving the quality of genome assemblies through curation. *GigaScience.* 2021;10(1):giaa153. 33420778 10.1093/gigascience/giaa153 PMC7794651

[ref24] Johnson AS Sebens KP : Consequences of a flattened morphology: Effects of flow on feeding rates of the scleractinian coral *Meandrina meandrites.* *Mar. Ecol. Prog. Ser.* 1993;99:99–114. 10.3354/meps099099

[ref25] Kerpedjiev P Abdennur N Lekschas F : HiGlass: Web-based visual exploration and analysis of genome interaction maps. *Genome Biol.* 2018;19(1):125. 30143029 10.1186/s13059-018-1486-1 PMC6109259

[ref26] Kurtzer GM Sochat V Bauer MW : Singularity: Scientific containers for mobility of compute. *PLOS ONE.* 2017;12(5):e0177459. 28494014 10.1371/journal.pone.0177459 PMC5426675

[ref27] Kwong WK Irwin NAT Mathur V : Taxonomy of the apicomplexan symbionts of coral, including Corallicolida ord. Nov., reassignment of the genus *Gemmocystis*, and description of new species *Corallicola aquarius* gen. Nov. Sp. Nov. And *Anthozoaphila gnarlus* gen. Nov. Sp. nov. *J. Eukaryot. Microbiol.* 2021;e12852. 33768669 10.1111/jeu.12852

[ref28] LaJeunesse TC Parkinson JE Gabrielson PW : Systematic revision of Symbiodiniaceae highlights the antiquity and diversity of coral endosymbionts. *Curr. Biol.* 2018;28(16):2570–2580.e6. 30100341 10.1016/j.cub.2018.07.008

[ref29] Li H : Minimap2: Pairwise alignment for nucleotide sequences. *Bioinformatics.* 2018;34(18):3094–3100. 29750242 10.1093/bioinformatics/bty191 PMC6137996

[ref30] Manni M Berkeley MR Seppey M : BUSCO update: Novel and streamlined workflows along with broader and deeper phylogenetic coverage for scoring of eukaryotic, prokaryotic, and viral genomes. *Mol. Biol. Evol.* 2021;38(10):4647–4654. 34320186 10.1093/molbev/msab199 PMC8476166

[ref31] Meesters EH Bak RPM : Effects of coral bleaching on tissue regeneration potential and colony survival. *Mar. Ecol. Prog. Ser.* 1993;96(2):189–198. 10.3354/meps096189

[ref32] Merkel D : Docker: Lightweight Linux containers for consistent development and deployment. *Linux J.* 2014;2014(239):2. 10.5555/2600239.2600241

[ref33] NOAA Florida Keys National Marine Sanctuary: Stony coral tissue loss disease case definition. 2018;10. Reference Source

[ref34] O’Neil KL Serafin RM Patterson JT : Repeated ex situ spawning in two highly disease susceptible corals in the family Meandrinidae. *Front. Mar. Sci.* 2021;8:669976. 10.3389/fmars.2021.669976

[ref35] Olm MR Brown CT Brooks B : dRep: A tool for fast and accurate genomic comparisons that enables improved genome recovery from metagenomes through de-replication. *ISME J.* 2017;11(12):2864–2868. 28742071 10.1038/ismej.2017.126 PMC5702732

[ref36] Parks DH Imelfort M Skennerton CT : CheckM: Assessing the quality of microbial genomes recovered from isolates, single cells, and metagenomes. *Genome Res.* 2015;25(7):1043–1055. 25977477 10.1101/gr.186072.114 PMC4484387

[ref37] Pinzón JH Weil E : Cryptic species in the Atlantic-Caribbean scleractinian genus *Meandrina*: A multi-variable review of the taxonomy and description of the new species *Meandrina jacksoni.* *Bull. Mar. Sci.* 2011;87(4):823–853. 10.5343/bms.2010.1085

[ref38] Precht WF Gintert BE Robbart ML : Unprecedented disease-related coral mortality in southeastern Florida. *Sci. Rep.* 2016;6:31374. 27506875 10.1038/srep31374 PMC4979204

[ref39] Ranallo-Benavidez TR Jaron KS Schatz MC : GenomeScope 2.0 and Smudgeplot for reference-free profiling of polyploid genomes. *Nat. Commun.* 2020;11(1):1432. 32188846 10.1038/s41467-020-14998-3 PMC7080791

[ref40] Rao SSP Huntley MH Durand NC : A 3D map of the human genome at kilobase resolution reveals principles of chromatin looping. *Cell.* 2014;159(7):1665–1680. 25497547 10.1016/j.cell.2014.11.021 PMC5635824

[ref41] Rhie A McCarthy SA Fedrigo O : Towards complete and error-free genome assemblies of all vertebrate species. *Nature.* 2021;592(7856):737–746. 33911273 10.1038/s41586-021-03451-0 PMC8081667

[ref42] Rhie A Walenz BP Koren S : Merqury: Reference-free quality, completeness, and phasing assessment for genome assemblies. *Genome Biol.* 2020;21(1):245. 32928274 10.1186/s13059-020-02134-9 PMC7488777

[ref43] Rosales SM Huebner LK Clark AS : Bacterial metabolic potential and micro-eukaryotes enriched in stony coral tissue loss disease lesions. *Front. Mar. Sci.* 2022;8:776859. 10.3389/fmars.2021.776859

[ref44] Rosales SM Huebner LK Evans JS : A meta-analysis of the stony coral tissue loss disease microbiome finds key bacteria in unaffected and lesion tissue in diseased colonies. *ISME Communications.* 2023;3(1):19. 36894742 10.1038/s43705-023-00220-0 PMC9998881

[ref45] Rühlemann MC Wacker EM Ellinghaus D : MAGScoT: A fast, lightweight and accurate bin-refinement tool. *Bioinformatics.* 2022;38(24):5430–5433. 36264141 10.1093/bioinformatics/btac694 PMC9750101

[ref46] Sandeman IM : Fine banding in the septa of corals. *Proceedings of the 11th International Coral Reef Symposium.* 2008;57–61.

[ref47] Schoch CL Ciufo S Domrachev M : NCBI taxonomy: A comprehensive update on curation, resources and tools. *Database.* 2020;2020:baaa062. 32761142 10.1093/database/baaa062 PMC7408187

[ref48] Seemann T : Prokka: Rapid prokaryotic genome annotation. *Bioinformatics.* 2014;30(14):2068–2069. 24642063 10.1093/bioinformatics/btu153

[ref49] Steiner SCC Kerr JM : Stony corals in Dominica during the 2005 bleaching episode and one year later. *Rev. Biol. Trop.* 2008;56(S1):139–148. 10.15517/rbt.v56i0.5583

[ref50] Uliano-Silva M Ferreira JGRN Krasheninnikova K : MitoHiFi: A Python pipeline for mitochondrial genome assembly from PacBio high fidelity reads. *BMC Bioinformatics.* 2023;24(1):288. 37464285 10.1186/s12859-023-05385-y PMC10354987

[ref51] Upton SJ Peters EC : A new and unusual species of coccidium (Apicomplexa: Agamococcidiorida) from Caribbean scleractinian corals. *J. Invertebr. Pathol.* 1986;47(2):184–193. 10.1016/0022-2011(86)90045-5

[ref52] Meij SET van der : Host species, range extensions, and an observation of the mating system of Atlantic shallow-water gall crabs (Decapoda: Cryptochiridae). *Bull. Mar. Sci.* 2014;90(4):1001–1010. 10.5343/bms.2014.1017

[ref53] Vasimuddin M Misra S Li H : Efficient architecture-aware acceleration of BWA-MEM for multicore systems. *2019 IEEE International Parallel and Distributed Processing Symposium (IPDPS).* IEEE;2019;314–24. 10.1109/IPDPS.2019.00041

[ref54] Zhou C McCarthy SA Durbin R : YaHS: Yet another Hi-C scaffolding tool. *Bioinformatics.* 2023;39(1):btac808. 36525368 10.1093/bioinformatics/btac808 PMC9848053

