# Supplementary material for: The chromosomal genome sequence of the maze coral, Meandrina meandrites (Linnaeus, 1758) (Scleractinia: Meandrinidae) and its associated microbial metagenome sequences
Source: Wellcome Open Res. 2026 Jul 28;11:469. [Version 1] doi: 10.12688/wellcomeopenres.27184.1 (PMC13560824; doi:10.12688/wellcomeopenres.27184.1)
Supplement: Peer review history

# Reviewer report: Reviewer response for version 1

**DOI:** 10.21956/wellcomeopenres.29923.r163212

**Published:** 2026-10-05

**Authors:** Jayan Duminda M Senevirathna

**Affiliations:** 1 Uva Wellassa University, Badulla, Uva Province, Sri Lanka

The authors have successfully generated a genome assembly for *Meandrina meandrites* (maze coral; Cnidaria; Anthozoa; Scleractinia; Meandrinidae), achieving approximately 96% BUSCO completeness. The genome has been assembled to the scaffold level, with the identification of pseudochromosomal structures, representing an important contribution to the genomic resources available for this species. Furthermore, the annotation of approximately 30,464 protein-coding genes provides a substantial and valuable genomic resource for future studies on the biology, evolution, population genetics, and conservation of *M. meandrites*. An additional strength of the study is that the genomic data have been made publicly available, facilitating their accessibility and reuse by the scientific community.

 One point that requires clarification is the assembly level reported in Table 2. The table currently indicates that the assembly is at the “chromosome level”; however, based on the information provided in the manuscript, the genome appears to have been assembled to the scaffold level, with pseudochromosomal structures subsequently identified or inferred. I therefore recommend that the authors verify and, if necessary, revise the terminology in Table 2 to accurately distinguish between scaffold-level assembly and chromosome/pseudochromosome-level organization.

 Overall, the genome assembly and annotation provide a valuable genomic resource for *M. meandrites* and represent a useful contribution to coral genomics. Subject to clarification of the assembly-level terminology in Table 2, I consider the manuscript suitable for acceptance.

Are sufficient details of methods and materials provided to allow replication by others?

Yes

Is the rationale for creating the dataset(s) clearly described?

Yes

Are the datasets clearly presented in a useable and accessible format?

Yes

Are the protocols appropriate and is the work technically sound?

Yes

Reviewer Expertise:

Aquatic Genetics and Biotechnology

I confirm that I have read this submission and believe that I have an appropriate level of expertise to confirm that it is of an acceptable scientific standard.

# Reviewer report: Reviewer response for version 1

**DOI:** 10.21956/wellcomeopenres.29923.r163209

**Published:** 2026-08-25

**Authors:** Adrián Jaimes-Becerra

**Affiliations:** 1 Tohoku University, Japan, Japan

This is a valuable chromosome-scale coral genome resource, and the central host-genome assembly is technically strong. The study presents a 551.16 Mb primary assembly for *Meandrina meandrites*, with 99.25% assigned to 14 chromosomal pseudomolecules, a scaffold N50 of 39.93 Mb, and 96.4% complete metazoa_odb10 BUSCOs. Accession numbers are provided for the raw PacBio HiFi, Hi-C, and RNA-seq data, and both the primary and alternate assemblies have been deposited in public repositories. The manuscript is also timely because *M. meandrites* is classified as Critically Endangered and is highly susceptible to stony coral tissue loss disease.

 I have only a few comments that I think could help improve the manuscript.

 1. The metagenome data are publicly discoverable through the ENA umbrella project PRJEB69492, which lists component project PRJEB86272: “ *Meandrina meandrites* (maze coral) coral metagenome assembly, jaMeaMean2.metagenome.” However, PRJEB86272 is not cited directly in the Data availability section or Table 5. In addition, the two rows in Table 5 are not linked to specific deposited records or sequence identifiers. I recommend citing PRJEB86272 directly and mapping each reported bin to its corresponding deposited identifier.

 2. The Methods state that MetaMDBG was used to generate the metagenome assembly and that “the resulting bin sets of each binning algorithm” were optimized and refined using MAGScoT. However, the individual binning algorithms are not identified. The manuscript also does not specify which PacBio HiFi reads, or which subset of reads, were used as input for MetaMDBG.

 3. You could explain why the first bacterial bin was not classified as a MAG, despite being reported as a single circular contig with 98.92% completeness and 0% contamination. 

 4. The relationship between the GenomeScope genome-size estimate and the final primary assembly span should be discussed. 

 5. Although the methods are described in considerable detail, and recognizing the format generally used for these Data Notes, the manuscript would benefit from a diagram overview of the complete experimental and bioinformatic workflow. 

 6. The manuscript states that size selection and cleanup were performed using diluted AMPure PB beads. Please report the AMPure PB bead dilution ratio and the final bead-to-sample ratio.

Are sufficient details of methods and materials provided to allow replication by others?

Partly

Is the rationale for creating the dataset(s) clearly described?

Yes

Are the datasets clearly presented in a useable and accessible format?

Yes

Are the protocols appropriate and is the work technically sound?

Yes

Reviewer Expertise:

Evolutionary biology; cnidarian biology; venom evolution; genomics, transcriptomics, and other omics approaches.

I confirm that I have read this submission and believe that I have an appropriate level of expertise to confirm that it is of an acceptable scientific standard.

# Reviewer report: Reviewer response for version 1

**DOI:** 10.21956/wellcomeopenres.29923.r163214

**Published:** 2026-08-19

**Authors:** Yichen Dai

**Affiliations:** 1 Fudan University, Shanghai, China

This Data Note presents a high-quality chromosome-level genome assembly for the maze coral *Meandrina meandrites* , a western Atlantic coral species that is highly susceptible to Stony Coral Tissue Loss Disease (SCTLD). The authors report a high quality genome assembly (551.16 Mb across 14 chromosomes) with excellent completeness metrics (96.4% BUSCO). The work addresses an urgent need for genomic resources for threatened coral species. I have several minor concerns and suggestions for improvement.

**1. Metagenome**

 The authors state that they "recovered two bins from the metagenome assembly, which did not meet the criteria for MAGs" (Metagenome report section). There is no discussion any further about taxonomic identity, completeness, or contamination levels of these bins. However, the metagenome component is prominently featured in the title. I suggest that the authors discuss whether these bins represent known coral-associated microbes, potential pathogens, or novel lineages, or remove the metagenome part from the title.

****2. Alternate** **h** **aplotype** **assembly and** ******k****** **-mer completeness****

 I’m slightly confused about which version is the "final assembly". Aside from the primary assembly, the authors also deposited an alternate haplotype assembly (GCA_963693325.1). The combined assembly has a *k* -mer completeness of 97.85% (primary + alternate), but the primary alone has only 75.79% completeness (Assembly quality metrics section). This suggests substantial allelic diversity is represented in the alternate haplotype. Perhaps the authors can discuss whether they think they have generated a true haplotype-resolved assembly worth further analysis, or an artefact of the assembly process that was retained due to high heterozygosity.

 In addition, this raises questions about whether researchers using only the primary assembly (which most will) will have sufficient representation of allelic diversity for population genomic and variant calling analyses. When the authors move on to discuss BUSCO values etc., are they using the combined assembly or primary assembly? I suggest that the authors add a sentence  explicitly addressing the implications of the 75.79% primary *k* -mer completeness for downstream applications. This is particularly important given the species' threatened status and the need for conservation genomics. I also suggest that the authors clearly state whether the combined assembly or primary assembly has been used for BUSCO evaluation and gene annotation.

****3** **.** **Implications for** **c** **onservation** **?****

 While the introduction provides an excellent background on the species' biology and SCTLD susceptibility, the genome assembly is not explicitly linked to specific research questions that the community can now address. The authors mention SCTLD repeatedly, but there is no discussion of how this genome will facilitate pathogen detection, host response studies, or conservation efforts. Perhaps the authors can mention briefly how this genome assembly may facilitate further research, for example identification of immune-related genes implicated in SCTLD susceptibility using comparative genomics.

****Minor** **c** **omments****

 1. The software versions table (Table 6) is comprehensive, but some versions appear outdated (e.g., CheckM 2015-01-16 is nearly a decade old; CheckM2 is now available). Can the authors justify the use of older tools or note if this was necessary for the coral metagenome data?

 2. The mitochondrial genome (17.2 kb) was assembled using *Diploastrea heliopora* as a reference. However, the authors mention that the "mitochondrial genome sequence of M. meandrites was recently published" (Baeza & Rosales, 2025). Is it worth the effort to quickly compare the authors' mitochondrial assembly with the published one? Are they identical?

 3. Table 2 heading "meandrina meandrines" should be " *Meandrina meandrites* ".

Are sufficient details of methods and materials provided to allow replication by others?

Yes

Is the rationale for creating the dataset(s) clearly described?

Yes

Are the datasets clearly presented in a useable and accessible format?

Yes

Are the protocols appropriate and is the work technically sound?

Yes

Reviewer Expertise:

Genome assembly; Comparative genomics

I confirm that I have read this submission and believe that I have an appropriate level of expertise to confirm that it is of an acceptable scientific standard, however I have significant reservations, as outlined above.

# Reviewer report: Reviewer response for version 1

**DOI:** 10.21956/wellcomeopenres.29923.r163213

**Published:** 2026-08-19

**Authors:** Poppy Hesketh-Best

**Affiliations:** 1 Foundation Institute of Research in Health Sciences Germans Trias i Pujol, Badalona, Catalonia, Spain

This this data note, the authors report the whole-genome sequencing of *Meandrina meandrites*, or maze coral. The capacity of *M. meandrites* to tolerate thermal stress makes it a valuable genome for study, particularly a specimen that has not been impacted by stony coral tissue loss disease.

**Is the rationale for creating the dataset(s) clearly described?**

 The rationale is appropriate for the genome announcement paper.

 - A citation to a report, peer-reviewed article, or survey is needed to support the statement that "no apparent SCTLD-affected corals were found" in the *Sample Acquisition* section. This is particularly important given that widespread SCTLD impacts across the region by 2019/2020 (Toth et al., 2022). The statement that no visibly affected corals were present at the sampling site requires additional support, specially given your sampling dates (2019).

**Are the protocols appropriate and is the work technically sound?**

 The protocols appear appropriate and are justified where necessary.

**Are sufficient details of methods and materials provided to allow replication by others?**

 The protocols are well described and are supported by references to highly detailed protocols available on Protocols.io and complete version controls are provided. There is some repetition throughout this section that could be streamlined.

 - First mention of Qubit device does not include version (under *Nuclear acid extraction*), only the third mention (under *PacBio Hi-Fi lib..*). "Qubit Fluorometer v4.0 (Thermo Fisher Scientific)" is also repeated twice times, unless it is a different version, this is unnecessary.

**Are the datasets clearly presented in a useable and accessible format?**

 The datasets and workflows are all publicly available and accessible (checked 2026-08-18)

**Minor comments:**

 - The abstract needs more detail regarding the recovered metagenomics bins. That final sentence is too brief. Unsure what the character/word limit is, but a little more should be stated.

 - Why is one of the bacterial bins classified as medium quality, and which criterion did it fail to meet? The single-contig circular genome with 98% completeness/0% contamination. If the medium-quality designation is due to the absence of required features (5/16/23S, tRNAs), this should be  stated in the **Metagenome Report **subsection.

Are sufficient details of methods and materials provided to allow replication by others?

Yes

Is the rationale for creating the dataset(s) clearly described?

Yes

Are the datasets clearly presented in a useable and accessible format?

Yes

Are the protocols appropriate and is the work technically sound?

Yes

Reviewer Expertise:

Microbial genomics

I confirm that I have read this submission and believe that I have an appropriate level of expertise to confirm that it is of an acceptable scientific standard.

## References

[1] : Stony coral tissue loss disease accelerated shifts in coral composition and declines in reef accretion potential in the Florida Keys. *Frontiers in Marine Science* .2023;10: 10.3389/fmars.2023.1276400 10.3389/fmars.2023.1276400
